# Combinatorial Approaches for Efficient Design of Photoswitchable Protein-Protein Interactions as *In Vivo* Actuators

**DOI:** 10.3389/fbioe.2022.844405

**Published:** 2022-02-08

**Authors:** Xiao Zhang, Yuxin Pan, Shoukai Kang, Liangcai Gu

**Affiliations:** Department of Biochemistry and Institute for Protein Design, University of Washington, Seattle, WA, United States

**Keywords:** combinatorial protein library, light induced dimerization, chemically induced dimerization, photoreceptor, optogenetics, actuator, opto-binder, nanobody

## Abstract

Light switchable two-component protein dimerization systems offer versatile manipulation and dissection of cellular events in living systems. Over the past 20 years, the field has been driven by the discovery of photoreceptor-based interaction systems, the engineering of light-actuatable binder proteins, and the development of photoactivatable compounds as dimerization inducers. This perspective is to categorize mechanisms and design approaches of these dimerization systems, compare their advantages and limitations, and bridge them to emerging applications. Our goal is to identify new opportunities in combinatorial protein design that can address current engineering challenges and expand *in vivo* applications.

## Introduction

The control of molecular proximity by protein-protein interactions (PPIs) is a fundamental mechanism in biology. It is widely employed in signal transduction, regulation of gene expression, metabolism, and other processes. The ability to noninvasively control PPIs *in vivo* with sufficient sensitivity and specificity would have numerous biomedical applications, for example, the site- and time-specific activation of therapeutic immune cells in solid tumors. In chemogenetic and optogenetic approaches, PPIs are controlled by chemicals [e.g., chemically-induced dimerization (CID) (Spencer et al., 1993; Stanton et al., 2018)] and light [e.g., light-induced dimerization (Shimizu-Sato et al., 2002; Klewer and Wu, 2019)], respectively. Among those, photoswitchable PPI systems are superior in spatiotemporal resolution and are not limited by toxicity and delivery issues associated with chemical inducers. Compared with single-component actuators such as microbial opsins (Zhang et al., 2011), the PPI systems provide the flexibility to regulate diverse biological processes at various subcellular locations (Stanton et al., 2018; Klewer and Wu, 2019).

Photoswitchable PPI systems function via different mechanisms with specific applications. A typical example is photoswitchable heterodimerization of a photoreceptor and a dimerization protein, which are genetically fused to proteins of interest to control the proximity. Some commonly used systems include phytochrome (Shimizu-Sato et al., 2002; Chernov et al., 2017), cryptochrome (Kennedy et al., 2010; Losi et al., 2018), and light-oxygen-voltage (LOV)-sensing-based dimerization systems (Strickland et al., 2012; Guntas et al., 2015; Kawano et al., 2015; Pudasaini et al., 2015). For specific applications, critical parameters to consider are activation wavelength, light sensitivity, the affinity, specificity, kinetics, and reversibility of dimerization, and expression compatibility in host cells. Due to the limited understanding of phototransduction in these proteins, it has been challenging to rationally design them for particular needs. Another important example is photoswitchable protein binders, hereafter named *opto-binders*, which can target unmodified endogenous proteins. A first opto-binder was a split green fluorescent protein (GFP) antibody comprising two fragments genetically tagged with a photoswitchable heterodimerization system (Yu et al., 2019). Like custom antibodies, opto-binders with defined binding properties are needed for a growing list of endogenous targets and for different cell types. However, there lacks an efficient, generalizable approach for creating such binders against arbitrary targets. The last but not the least example is *photocaged drug-controlled CID* which is independent of a photoreceptor. This mechanism is exemplified by an emerging class of protein degradation drugs, known as photoswitchable proteolysis targeting chimeras (photoPROTACs) (Pfaff et al., 2019). Presumably, photo-uncaging of drugs can be used to simultaneously control their pharmacologic effects and the CID-regulated other outcomes. However, demonstrating the full potential of this actuation mechanism relies on the ability to create CID systems for a variety of drugs of interest.

The complex physicochemical properties and conformational dynamics of photoswitchable PPI systems impose challenges on the *de novo* design, but some successes were recently achieved by screening combinatorial protein libraries (Jang and Woolley, 2021). This perspective aims to build upon the initial success to identify new opportunities to break existing barriers in the protein design. It is worth noting that PPI systems have different degrees of complexity (e.g., homo- or hetero-assemblies with two or more protein components). Here, we focus on heterodimerization systems which in our opinion are the simplest but most convenient actuator device for engineering and applications. We will discuss the three major photoswitchable dimerization mechanisms, current challenges in their design, and then propose combinatorial approaches to address them.

### Photoswitchable Heterodimerization

**FIGURE 1:**
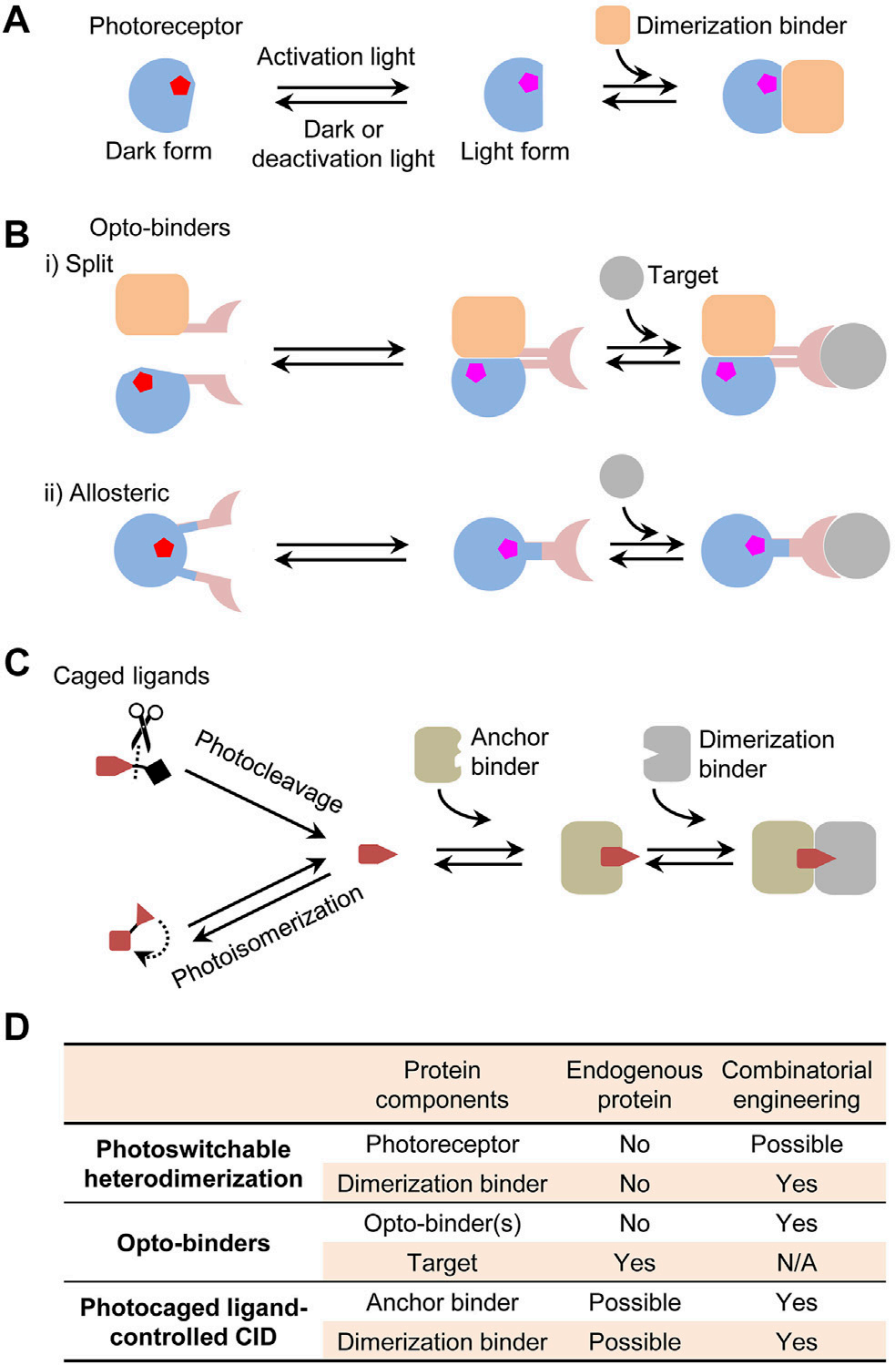
Photoswitchable protein dimerization mechanisms. **(A)** Photoswitchable heterodimerization. Pentagons indicate chromophores in different conformations (red or magenta). Although a dimerization binder might specifically recognize the light or dark form, only the binding to the light form is shown. **(B)** Opto-binders. Split and allosteric opto-binders function via the protein-fragment complementation and allosteric mechanisms, respectively. **(C)** Photocaged ligand-controlled CID. The uncaged ligand first binds to an anchor binder and then the anchor binder-ligand complex, not the free anchor binder, is recognized by a dimerization binder. **(D)** Comparison of protein components in the dimerization systems regarding whether they can be an endogenous protein and whether they can be engineered by a combinatorial approach. N/A, not applicable.

A typical heterodimerization system comprises a photoreceptor protein or domain that undergoes a conformational switch between a light and a dark form and a dimerization binder recognizing the conformational change (Figure 1A). For actuation applications, photoreceptors and dimerization binders are fused to proteins of interest to exert the proximity control (Levskaya et al., 2009; Kennedy et al., 2010). Their applications have benefited from the availability of naturally occurring photoreceptors activated by a broad range of wavelengths from ultraviolet to near-infrared (NIR). These photoreceptors have been thoroughly discussed in the most recent reviews (Farahani et al., 2021; Manoilov et al., 2021; Seong and Lin, 2021) and are not the focus of this perspective. A broad selection of optical inputs enables advanced biological actuation such as controlling multiple cellular events with different wavelengths (Redchuk et al., 2017; Ochoa-Fernandez et al., 2020) and activating gene expression in deep tissues with NIR light (Kaberniuk et al., 2016). However, natural photoreceptors and dimerization binders often have extra domains irrelevant to photosensory and dimerization functions but with other important roles in their original hosts. In an actuator, they can cause unwanted interference to cell signaling and difficulties for mammalian expression or gene delivery due to the large gene sizes surpassing the packaging capacity of adeno-associated virus (Wu et al., 2010). Sometimes simply truncating them can disrupt the stability and function of the core photosensory and dimerization domains due to possible interdomain interactions (Bellini and Papiz, 2012). Additionally, the dimerization sometimes lacks adequate affinity or specificity, resulting in a low light activation sensitivity or a high dark activity. To overcome inherent limitations of the natural proteins, it is necessary to *de novo* engineer components of the systems such as dimerization binders.

### Opto-Binders

Some applications require controlling unmodified endogenous proteins rather than those genetically tagged with photoreceptors or dimerization binders. This became possible by using opto-binders derived from a binder of an endogenous target. Two types of opto-binders were demonstrated based on protein-fragment complementation and allosteric mechanisms (Figure 1B). The first type is similar to a protein-fragment complementation assay (Pelletier et al., 1998) in which, upon light activation, two fragments of a split binder tagged with a photoswitchable dimerization system are brought in close proximity to form a functional unit (Yu et al., 2019). An opto-binder can also be designed by inserting a photoreceptor domain into an allosteric site of a binder [e.g., nanobody (Gil et al., 2020; He et al., 2021), monobody (Carrasco-López et al., 2020; He et al., 2021), and affibody (Woloschuk et al., 2021)] to regulate the binding activity. By comparison, single-component allosteric opto-binders are easier to use than split opto-binders because it is more difficult to control expression levels and binding behaviors of two-component than single-component systems in cells. However, the design of the allostery in opto-binders had limited success and typically relied on a slow and costly trial-and-error process to select photoreceptors, binders, insertion sites, and linkers to create a chimeric fusion.

### Photocaged Ligand-Controlled CID

Some dimerization systems do not require a photoreceptor but instead use a photocaged ligand to control a CID system. Photo-uncaging of the ligand induces the dimerization of two proteins termed as anchor and dimerization binders (Figure 1C). This mechanism was first demonstrated with an engineered CID system induced by a photocleavage analogue of rapamycin (Karginov et al., 2011) and reviewed in the previous literature (Ankenbruck et al., 2018). For *in vivo* regulation, CID systems offer better flexibility than the photoreceptor-dependent dimerization because unmodified endogenous proteins can possibly serve as an anchor binder [e.g., ligand-induced G protein-coupled receptor–nanobody complex (Kruse et al., 2013)] or both an anchor and a dimerization binders [e.g., PROTAC-induced E3 ligase–target complexes (Sakamoto et al., 2001)] (Figure 1D). There could be a wide choice of photocaged CID inducers derived from natural or synthetic drugs, metabolites, or other molecules by introducing a photocleavable [e.g., coumarin and 2-nitrobenzyl derivatives (Klán et al., 2013)] or photoisomerizable moiety [e.g., azobenzenes (Beharry and Woolley, 2011)]. Compared with other dimerization mechanisms, a distinct advantage of these systems is the dual-control by chemical and light where a drug inducer can potentially exert both the pharmacological and CID-regulated effects. Although progress was made on designing ligands for some classes of proteins [e.g., photoPROTACs (Liu et al., 2020; Reynders et al., 2020)], a generalizable strategy is needed for creating CIDs for any arbitrary drug.

### *In Vitro* Screening of Combinatorial Protein Libraries

**FIGURE 2:**
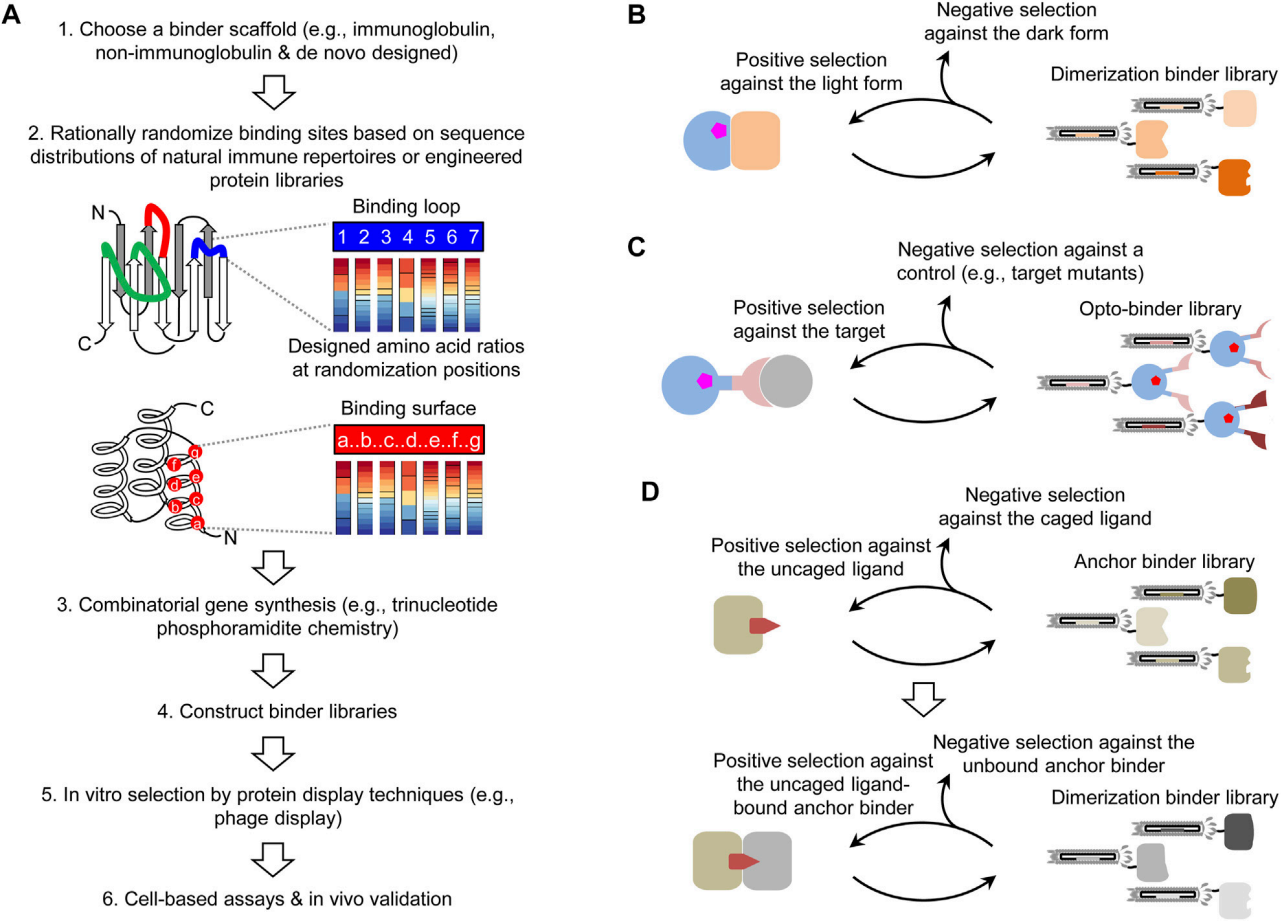
Combinatorial approaches for creating photoswitchable dimerization systems. **(A)** Pipeline of the design and screening of combinatorial binder libraries. **(B)** Combinatorial approach for obtaining dimerization binders in a photoswitchable heterodimerization system. **(C)** Selection of a combinatorial optobinder library constructed with a binder scaffold inserted with a photoreceptor for the allosteric regulation. The opto-binder library is applicable to arbitrary targets. **(D)** Generalizable, two-step approach for creating CID systems for an uncaged ligand.

A strategy to design photoswitchable dimerization is to use protein binders to sense light-induced conformational change(s). However, photoreceptors exhibit function-associated structural dynamics (Genick et al., 1998; Takala et al., 2014), imposing a major challenge for the structure-based binder design. Some conformational states of these proteins are difficult to stabilize to produce immunogens for animal immunization. To date, the *de novo* engineering of binders to an existing photoreceptor mainly relies on *in vitro* screening of combinatorial protein libraries. In this approach, a critical step is to design and synthesize a high-quality combinatorial library (Figure 2A). Like natural antibody repertoires, a synthetic combinatorial library is designed from a shared protein scaffold, e.g., an immunoglobulin (Maynard and Georgiou, 2000; Muyldermans, 2013), non-immunoglobulin (Yu et al., 2017; Gebauer and Skerra, 2020), or *de novo* designed scaffold. The scaffold is introduced with rationally randomized binding site(s), for example, variable flexible loops like antibody complementarity-determining regions or residues on a relatively rigid binding surface. To obtain a high percentage of protein sequences in the library that might be correctly translated and stably folded, the randomization is usually guided by amino acid distributions found in experimentally validated natural [e.g., antibody repertoires (Moutel et al., 2016)] or engineered binder libraries. A trinucleotide phosphoramidite chemistry (Virnekäs et al., 1994) is ideally suited to control codon ratios at randomization sites. The combinatorial gene synthesis can generate a vastly diverse library (>10^9^) with clones that can virtually bind to any given epitope. Such large libraries are suitable for protein display-based *in vitro* screening, e.g., phage (Smith, 1985), ribosome (Hanes and Plückthun, 1997), and mRNA display (Roberts and Szostak, 1997). An important advantage of the *in vitro* screening is that photosensitive reagents such as photoreceptors are easier to stabilize in light-conditioned assays. Based on our previous experience, an effective screening of a high-quality library can identify suitable conformation-selective binders even without further mutagenesis.

### Approach 1. Screening Dimerization Binders for Photoreceptors

The first application of combinatorial binder libraries is to select dimerization binders against the light or dark form of a photoreceptor to create heterodimerization systems. This approach can circumvent unavoidable constraints of natural dimerization binders and provide the flexibility to select the protein size and binding properties. Typically, dimerization binders are obtained by screening a combinatorial library against the light or dark form of a photoreceptor as the positive selection and the other form as the negative selection (Figure 2B). Candidates with various binding parameters are chosen or further engineered for specific applications. It might be possible to design combinatorial photoreceptor libraries (e.g., surface mutants), but it is necessary to assess how these mutations will impact the photoconversion, for example, by a deep mutational scanning analysis (Fowler and Fields, 2014).

Initial successes were demonstrated by screening libraries of small size proteins against a few photosensory domains. Combinatorial libraries of affibodies [∼6 kDa (kDa)] were screened by mRNA and ribosome display against a dark-form LOV2 domain of *Avena Sativa* phototropin 1 (*As*LOV2) (Wang et al., 2016) and a light-form photosensory module of a *Deinococcus radiodurans* bacterial phytochrome (*Dr*BphP) (Kuwasaki et al., 2021) to obtain the light-induced dissociation and dimerization, respectively. Other dimerization systems were obtained by phage display screening of a surface mutant library of an albumin-binding domain (∼5 kDa) against circularly permuted photoactive yellow protein (PYP) (Reis et al., 2018) and a cGMP phosphodiesterase-adenylate cyclase-FhlA (GAF) domain of *Acryochloris marina* cyanobacteriochrome (CBCR) (Jang et al., 2019). We recently demonstrated an efficient method, COMBINES-LID, by screening a nanobody library to create red light-switchable actuators (Huang et al., 2020). In this work, a widely used library of nanobodies (∼12–15 kDa, a diversity of 1.23–7.14 × 10^9^) with a humanized scaffold was screened by phage display against the *Dr*BphP photosensory module. Column-based biopanning was designed for the selection of both the conformational specificity and dimerization reversibility. The library screening was demonstrated to be highly efficient; *in vivo* actuators constructed with selected dimerization binder clones showed drastically improved light-induction specificity and a significantly lower dark activity than a similar natural *Rhodopseudomonas palustris* BphP1 (RpBphP1) system and its derivative. Although the protein display screening is efficient, it is necessary to validate the expression of *in vitro* selected binders by cell-based assays [e.g., yeast (Fields and Song, 1989) or mammalian-two hybrid (Lievens et al., 2009)]. Based on these results, we propose that this approach should be applicable to other photoreceptors and combinatorial libraries for designing actuators with different structural, optical, and biochemical properties.

### Approach 2. Screening Combinatorial Opto-Binders for Endogenous Proteins

Beyond standard binder libraries, it is possible to insert a photosensory domain into a binder scaffold to design a combinatorial opto-binder library. Photoreceptor-regulated allostery was engineered to control enzyme activities (Reynolds et al., 2011; Richter et al., 2016) and recently demonstrated in creating opto-binders. For opto-binders derived from nanobodies (Gil et al., 2020; He et al., 2021) and monobodies (Carrasco-López et al., 2020; He et al., 2021), a truncated *As*LOV2 domain was inserted into specific loops away from their target binding sites, so the light-triggered unfolding of the Jɑ helix in *As*LOV2 allosterically regulates the binding site conformation. In the affibody-based opto-binders (Woloschuk et al., 2021), the PYP was inserted into a loop connecting two helices important to the affibody stabilization and target binding, resulting in mutually exclusive folding of the affibody and PYP. Thus, the light-induced partial unfolding of PYP drives the affibody binding to its target. This approach of converting a known binder to opto-binders for the same target is error prone because the photoreceptor insertion tends to change or disrupt the binding to the target even without the allosteric regulation (Mathony and Niopek, 2021). Thus, the current opto-binder design from a known binder is relatively inefficient. Alternatively, we propose to directly screen a combinatorial opto-binder library against a target. Given that photoreceptor insertion and target binding sites are different in successful opto-binders, it is possible to use them as scaffolds and implement a similar binding site randomization protocol to generate combinatorial opto-binder libraries. If it is successful, this new approach will greatly facilitate the generation of opto-binders targeting arbitrary endogenous proteins, interactions, and modifications.

The screening of opto-binder libraries can be performed with established methods such as COMBINES-LID (Huang et al., 2020). For example, to select opto-binders targeting a specific epitope in a target, an activation light-illuminated phage library will pass through a negative and a positive selection columns preloaded with an epitope mutant and the wildtype target, respectively (Figure 2C). Binders captured in the positive selection column will be eluted by deactivation light illumination or dark relaxation to select reversible opto-binders. Because it is unknown whether opto-binders can be functionally displayed by protein display techniques, it is necessary to test the efficiency of protein folding and chromophore incorporation for different opto-binder scaffolds.

### Approach 3. Screening CID Systems for Photo-Uncaged Ligands

Besides the photoreceptor-dependent dimerization, the combinatorial screening is applicable to engineering CID. CID systems have different designs for specific applications, but considering wide interest in optopharmacology, we focus on those whose chemical inducers are photo-uncaged drugs targeting endogenous proteins. Here, one or two binder components in these systems are selected from combinatorial libraries. Compared with other CIDs, this type of system provides three switches: 1) the photo-uncaging of drugs, 2) the binding of drugs to endogenous targets, and 3) the drug-induced dimerization. Although such CID systems are yet to be reported, based on available methods for the CID engineering (Kang et al., 2019) and chemical synthesis (Klán et al., 2013), it is possible to design them for many intriguing applications. For example, to spatiotemporally regulate therapeutic T cells, a drug can be light activated at a tumor site to mediate multiple cell signaling events: e.g., targeting endogenous proteins such as a programmed death ligand-1 (PD-L1) to inhibit the checkpoint signaling of tumor-dwelling T cells (Zak et al., 2016) and dimerizing T-cell receptor signaling proteins to activate the T cells (Wu et al., 2015).

To create such systems, a critical need is to design CID systems for any given drug. Recent advances were made by selecting dimerization binders from phage-displayed libraries of antigen-binding fragments (Fabs) (Hill et al., 2018) and nano-CLostridial antibody mimetic proteins (nanoCLAMPs) (Guo et al., 2021) against an existing drug–target complex. However, this method is limited to structure-defined drug–target complexes and cannot be applied to other drugs with unknown or unstable drug–target complexes. To address this limitation, we recently developed a two-step screening method (COMBINES-CID) to sequentially select anchor and dimerization binders from a combinatorial nanobody library (Figure 2D) (Kang et al., 2019). As a proof-of-concept, we chose a challenging drug inducer, cannabidiol (CBD) with low molecular weight and high hydrophobicity, and obtained CIDs with high ligand specificity. We have successfully applied this method to other combinatorial libraries and drug inducers (unpublished). To obtain CIDs only induced by photo-uncaged not caged drugs and without drug-independent auto-dimerization, the dimerization binder screening requires the negative selection using unbound anchor binders and caged drugs as controls. Presumably, the proposed approach can be integrated with a variety of photo-caged drugs to achieve the flexible, multimodal actuation.

## Discussion

The proposed combinatorial approaches will leverage ongoing efforts on designing photoswitchable dimerization systems for *in vivo* actuation needs. They can potentially generate functional data for large-scale protein sequences which are useful for developing deep learning methods (Cong et al., 2019; Humphreys et al., 2021) to improve computational PPI design. Beyond traditional screening methods, other technologies enabling the “library-by-library” screening [e.g., SMI-seq (Gu et al., 2014)] can facilitate the engineering of complex dimerization surfaces. Besides using photoreceptors to create actuators, other sensory proteins that convert chemical or physical signals to conformational changes [e.g., covalent modifications (Hoppmann et al., 2014), electric field (Sasaki et al., 2006), heat (Caterina et al., 1997), and mechanical force (Liedtke et al., 2003)] can also be used as targets for dimerization binder selection to create switchable dimerization systems. Overall, the methodology innovation and the sensory protein discovery will significantly expand the biosensor toolkit and revolutionize *in vivo* applications.

## Data Availability

The original contributions presented in the study are included in the article/supplementary material, further inquiries can be directed to the corresponding author.
